# Identification and characterisation of endogenous Avian Leukosis Virus subgroup E (ALVE) insertions in chicken whole genome sequencing data

**DOI:** 10.1186/s13100-020-00216-w

**Published:** 2020-06-30

**Authors:** Andrew S. Mason, Ashlee R. Lund, Paul M. Hocking, Janet E. Fulton, David W. Burt

**Affiliations:** 1 The Roslin Institute and Royal (Dick) School of Veterinary Studies, The University of Edinburgh, Easter Bush, Midlothian, EH25 9RG UK; 2 York Biomedical Research Institute, The Department of Biology, The University of York, York, YO10 5DD UK; 3 Hy-Line International, 2583 240th Street, Dallas Center, Iowa, 50063 USA; 4 The University of Queensland, Brisbane, Queensland 4072 Australia

**Keywords:** ALVE, Ev gene, Avian Leukosis virus, Endogenous retrovirus, obsERVer, Chicken

## Abstract

**Background:**

Endogenous retroviruses (ERVs) are the remnants of retroviral infections which can elicit prolonged genomic and immunological stress on their host organism. In chickens, endogenous Avian Leukosis Virus subgroup E (ALVE) expression has been associated with reductions in muscle growth rate and egg production, as well as providing the potential for novel recombinant viruses. However, ALVEs can remain in commercial stock due to their incomplete identification and association with desirable traits, such as ALVE21 and slow feathering. The availability of whole genome sequencing (WGS) data facilitates high-throughput identification and characterisation of these retroviral remnants.

**Results:**

We have developed obsERVer, a new bioinformatic ERV identification pipeline which can identify ALVEs in WGS data without further sequencing. With this pipeline, 20 ALVEs were identified across eight elite layer lines from Hy-Line International, including four novel integrations and characterisation of a fast feathered phenotypic revertant that still contained ALVE21. These bioinformatically detected sites were subsequently validated using new high-throughput KASP assays, which showed that obsERVer was highly precise and exhibited a 0% false discovery rate. A further fifty-seven diverse chicken WGS datasets were analysed for their ALVE content, identifying a total of 322 integration sites, over 80% of which were novel. Like exogenous ALV, ALVEs show site preference for proximity to protein-coding genes, but also exhibit signs of selection against deleterious integrations within genes.

**Conclusions:**

obsERVer is a highly precise and broadly applicable pipeline for identifying retroviral integrations in WGS data. ALVE identification in commercial layers has aided development of high-throughput diagnostic assays which will aid ALVE management, with the aim to eventually eradicate ALVEs from high performance lines. Analysis of non-commercial chicken datasets with obsERVer has revealed broad ALVE diversity and facilitates the study of the biological effects of these ERVs in wild and domesticated populations.

## Background

Retroviruses present persistent and unique challenges to the vertebrate species they infect. Exogenous retroviruses typically evolve at rates up to six orders of magnitude faster than their hosts and transfer novel accessory genes horizontally from other vertebrates, their parasites or other viruses [1–4]. Retroviruses also impact long term genome evolution as endogenous retroviruses (ERVs) following integration into the germline. ERVs represent a ‘fossil record’ of previous retroviral infections, and constitute approximately 3% of the avian genome [5, 6]. Identifiable avian ERVs include lineage-specific evolutionary ‘recent’ loci, integrations found across birds, and ancient sites also seen in mammalian genomes [5, 6].

ERVs decay, or are epigenetically silenced, over long evolutionary timescales [2, 3, 7, 8]. However, recurrent infection and intracellular retrotransposition can generate new, structurally intact ERVs with the potential to impact gene expression, facilitate chromosomal rearrangements and modulate host response to retroviral infections [4, 9–14]. ERVs may also continue to express their own retroviral genes (*gag*, *pol*, and *env*), driven by promoters with the potential for bidirectional effects in the flanking long terminal repeats (LTRs). Furthermore, they can re-emerge from the genome by recombination to pose novel exogenous threats, such as Avian Leukosis Virus (ALV) subgroup J in chickens [1, 4, 11, 13, 15, 16].

ALV is an alpharetrovirus which infects galliform birds, and is the only known chicken (*Gallus gallus*) retrovirus with both exogenous and endogenous activity [13, 17]. Exogenous ALVs are generally slow-transforming viruses which induce lymphoid (subgroups A-D) or myeloid (subgroup J) tumour formation over weeks or months via insertional mutagenesis, and can spread through flocks via viral shedding [18, 19]. Endogenous ALVs can also infect horizontally but have a species-specific range. Subgroup E (the ALVEs; historically known as *ev* genes) are found in the domestic chicken and its wild progenitor, the red jungle fowl (RJF), but in no other Gallus species [15, 20].

As evolutionarily recent integrations, ALVEs are typically found at low copy number in the genome, but often retain high structural integrity [21–23]. The presence of replication-competent ALVEs, and ALVEs which express gag proteins, has been associated with impacts on traits including reductions in muscle growth rate, egg number, size and shell thickness, and an increased incidence of viral shedding [24–28]. Conversely, *env* expression can mediate ALV infection through receptor interference [29–31]. As genomic ALVE content increases, their cumulative influence becomes increasingly complex, particularly when lines are interbred. Furthermore, recent work has shown interactions between ALVEs and the increasingly virulent Marek’s Disease Virus (MDV), a chicken-specific alphaherpesvirus. MDV can induce ALVE expression, even of normally silenced elements such as ALVE1 [32, 33], and MDV vaccines can induce higher incidence of spontaneous lymphoid tumours in lines containing ALVE21 [34–36].

Despite these effects, and the creation of ‘ALVE-free’ lines [37, 38], the commercial poultry breeding community has been unable to completely remove all ALVEs from breeding stock. This has been a combination of the inability to detect all ALVE insertions, the association between some ALVEs and commercially desirable traits (such as ALVE21 with slow feathering [39–43], and ALVE-TYR with white plumage [44, 45]), and managing selection programmes for multiple performance traits. Crucially, commercial breeding stock must be negative for exogenous ALV before shipment. This necessitates continual testing for the ALV-specific antigen p27, which generates an effective false positive when endogenous expression is detected. Gel-based PCR assays were developed to detect ALVEs common in layers [21], but such assays are uneconomical at commercial scales and were limited to known sites. Traditional ALVE identification methods utilised characteristic restriction fragment length polymorphisms (RFLPs), but these patterns were poorly conserved between breeds, and can be difficult to interpret when there are high ALVE numbers per bird [46–48]. Comprehensive ALVE identification and characterisation within commercial lines is therefore essential for improvements in both productivity and health monitoring, but any assays must be cost effective at commercial scales.

Beyond commercial chickens, little is known about ALVE diversity in other domesticated or wild chicken populations. The reference RJF assembly contains two ALVEs, one of which is commonly shared with commercial layers and broilers [21, 49, 50]. Short read, whole genome sequencing (WGS) datasets have been generated for many different chicken lines, breeds and populations (including wild-caught RJF) over the last decade, and present an opportunity for the identification of ALVEs. However, identification of such structural variants has been hindered by complex read mapping, incomplete reference genome assemblies and limited sequence coverage at insertion junction sites. Recent work has used target capture sequencing to enrich for repetitive DNA, including ALVEs [23, 51], to promote identification of novel integrations. However, these datasets have limited future applications, and most WGS data has not been mined fully for structural variants, including ERVs.

Here, we describe obsERVer, a bioinformatic pipeline developed for the detection of specific, user-determined ERV integrations in WGS datasets. We validated the bioinformatic predictions in eight elite layer lines with new high-throughput diagnostic assays for each identified ALVE, which we then used to genotype the originally sequenced birds and over 9000 archived DNA samples from the same elite layer lines. We also utilised obsERVer to identify ALVEs in fifty-seven diverse chicken datasets encompassing commercial, experimental and heritage layers and broilers, native breeds, and wild populations, including RJF. This work has enabled a better understanding of ALVEs in both commercial and more diverse chicken lines including the identification of over 260 novel ALVE loci. In addition, these methods provide new opportunities to examine the biological effects of ALVEs in diverse domesticated and wild chicken populations.

## Methods

### Whole genome sequencing datasets

A total of sixty-five Illumina paired-end 101 base pair (bp) chicken WGS datasets were surveyed for ALVE integrations. Datasets were available either from public repositories or kindly shared by collaborators, derived from individual birds or multiple individual pools as indicated in Table S1. These datasets included: experimental White Leghorn (WL), Brown Leghorn (BL) and Rhode Island White (RIW) lines; commercial layers (WL, White Plymouth Rock (WPR) and Rhode Island Red (RIR)); heritage broilers; native breeds and village populations from Asia and Africa; and wild-caught RJF from China, Java and Sumatra.

All sequencing reads were quality checked by FastQC v0.11.2 [52]. TrimGalore v0.4.0 [53] and Cutadapt v1.4 [54] were used to remove sequencing adapters and trim reads where base quality dropped below 20 in a 4 bp sliding window, removing reads trimmed by more than half their length. Each dataset was aligned to the Gallus_gallus5.0 reference genome (Galgal5; GenBank: GCF_000002315.4) using BWA-mem v0.7.10 [55], and coverage calculated with samtools v0.1.19 mpileup [56].

### Bioinformatic detection of ALVE integration sites

For detection of ALVE integrations, sequencing reads from each chicken WGS dataset were remapped to an “ALVE pseudochromosome” constructed of eleven publicly available ALV sequences (Table S2), each separated by 1 kilobase pair (kbp) of Ns (ambiguous bases). Reads which mapped to this ALVE pseudochromosome (and their read mates) were subtracted from the original FASTQ files for each dataset and then re-mapped against the Galgal5 reference genome to identify putative integration sites. Mapping quality [56] greater than 20 was required in both cases. This approach facilitated targeted analysis of user-defined integrations and reduced computational burden by first aligning to a limited reference sequence set rather than the entire genome, as in other recent analogous approaches [57, 58].

Assembled endogenous alpharetroviral sequences were detected by BLASTn [59] using publicly available ALV, EAV (endogenous avian virus) and ART-CH (avian retrotransposon of chicken) sequences (Table S2) and used to filter the list of putative integration sites. Remaining sites were filtered on the presence of split reads, where part of the read aligned to the reference genome and the remainder to an unassembled ALVE, precisely defining the integration site.

These scripts are contained within a single bioinformatics pipeline, obsERVer (Figure S1), available on GitHub (https://github.com/andrewstephenmason/obsERVer).

Putative ALVE integration sites were inspected in IGV Desktop for Windows v2.3.60 [60] and named using existing ALVE nomenclature where possible (Table S3). New names were given to novel insertions following the format “ALVE_ros001”, and to previously identified ALVEs with ambiguous names. Presence of the reference-genome-assembled ALVE-JFevA (ALVE6) and ALVE-JFevB integrations was checked manually in the full BAM files. ALVE orientation, terminal sequence integrity, and target site duplication (TSD) was identified using the split reads at each site. ALVEs which had integrated within other repeat elements were identified using RepeatMasker v4.0.3 [61].

Detection of ALVEs by obsERVer was developed using WGS datasets from eight Hy-Line (HL) elite layer lines (five independent WLs, sister WPRs, and one RIR), each derived from a pool of ten birds [62]. Following validation of the bioinformatically detected ALVE integrations (see below), obsERVer was implemented on the remaining WGS datasets.

### Validation of ALVEs detected by obsERVer

**Fig. 1:**
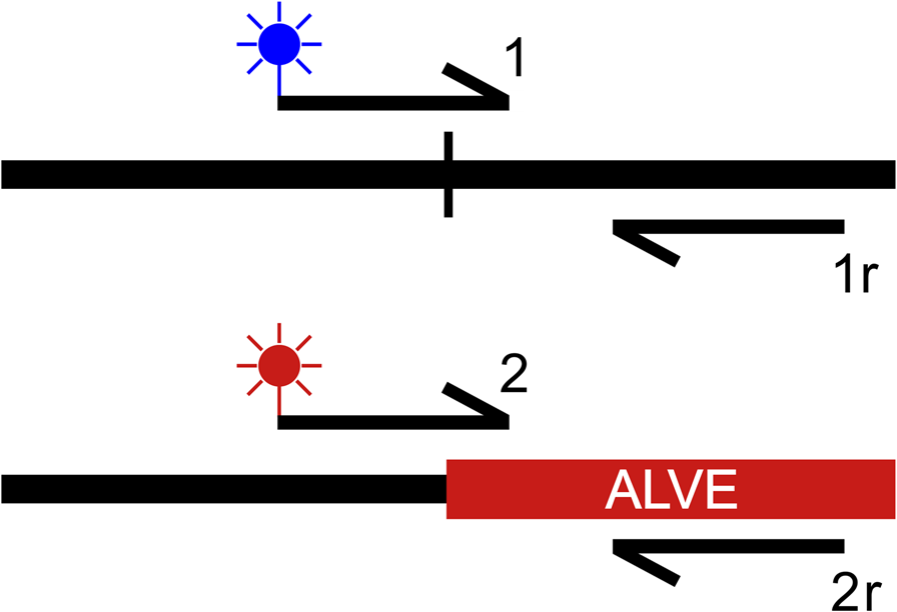
KASP assay primer design rationale for ALVE integration sites. Primer 1 (wildtype) and primer 2 (ALVE) are fluorophore-labelled primers and their amplification enables genotyping direct from solution. The starting sequence for the genotype-specific primers is often the same, but they differ when they cross the target site duplication, with primer 1 continuing through the host genome sequence and primer 2 entering the ALVE insertion. Rounds of elongation are short, so amplification between primer 2 and 1r would be unlikely, even with short insertions. Such short amplification required a four-primer approach rather than the single reverse primer typical in SNP genotyping KASP assays

Due to the complexities of structural variant detection from short read sequencing data, we assessed the number of false positives detected by obsERVer by validating the ALVEs detected in the HL WGS datasets. These datasets were the focus of obsERVer development and validation due to the availability of same-line DNA, including those from the original sequencing pools. Diagnostic assays were developed to validate each bioinformatically identified ALVE, as well as the *K* locus duplication site associated with ALVE21. Sequence from the ALVE-homologous split reads and the flanking genome at each putative integration site was used to develop KASP™ (Kompetitive Allele-Specific PCR; LGC, UK) assays using a four-primer approach (Fig. 1). Briefly, KASP assays use allele specific primers with fluorescent tags to directly genotype alternative alleles; enabling high-throughput detection of variants in a highly automated process. Initially designed for single nucleotide polymorphism (SNP)-genotyping, we have adapted the system to detect ALVE integrations.

Primers were designed with Kraken™ Primer Picker software (LGC, UK) with lengths of 20–25 bases and equal primer GC content in the 40–50% GC range (Table S4). Reactions used dehydrated DNA samples, primers and the LGC KASP™ 2x Mastermix V4.0 1536 formulation, following the original KBiosciences KASP™ protocol: a 61 °C to 55 °C touchdown for ten cycles and then 55 °C for twenty further cycles in a total reaction volume of 1 μl. Allele-specific fluorescence was detected using the PHERAstar Plus SNP plate reader and genotypes determined using Kraken™. For those ALVEs which had a previously reported PCR based test, we unambiguously validated genotype results between the KASP detection and gel-based detection method, thus confirming that we were detecting the same ALVE. All developed assays were used to genotype the original eighty individuals included in the sequencing pools (Table S1), as well as over 9000 banked samples from multiple generations of the eight sequenced lines.

In addition, traditional gel-based PCR assays were developed for those HL ALVEs that did not have previously published gel-based assays (Table S5). Primers were designed using Primer3 v2.3.7 [63].

### Modelling obsERVer detection sensitivity

The likelihood of missing ALVEs by chance from each HL dataset was modelled for all genotype frequencies given the pool size of ten individuals used for sequencing, total line population size, and the average observed genome coverage (11-18X). Mapping error rates were included based on the proportion of unmapped reads in each dataset, but the ‘sequenceability’ of the genome was not included due to limited literature on the estimation of this value [64–66]. For a given allele frequency, the model was run as follows: 1) a flock of given size was randomly assigned genotypes based on Hardy-Weinberg Equilibrium, 2) individuals and alleles were sampled according to a binomial distribution, and 3) then scaled for genome-average coverage and Poisson-varied coverage, both of which were further scaled by an underlying error rate. Models were run one million times. Probabilities were calculated for each sequenced HL line, for each possible insertion allele frequency. Probabilities were calculated for the HL ALVEs detected by KASP assay but not obsERVer, using the non-Poisson-scaled probabilities as site coverage was known.

### Sanger sequencing of ALVE integrations

KASP assays were used to identify individuals homozygous for each HL ALVE. ALVEs were amplified using the Takara PrimeSTAR® GXL DNA polymerase kit with the flanking genomic primers developed for the gel-based PCRs (Table S5). Amplification followed the standard Takara protocol with eight-minute extensions in each cycle.

DNA was extracted from excised gel bands using the Invitrogen PureLink™ Quick Gel Extraction kit. DNA from ALVE inserts larger than 1kbp was cloned into the Invitrogen ZeroBlunt TOPO pCR®4 Blunt-TOPO® vector and used to transform One Shot® Mach1™-T1^R^ Competent *E. coli* cells. Plasmids were extracted using the Invitrogen PureLink™ Quick Plasmid Miniprep kit following the manufacturer’s instructions. Purified DNA from ALVE inserts shorter than 1kbp was not cloned, but cleaned after PCR with the ExoSAP protocol.

Purified ALVE DNA was amplified for sequencing using the Applied Biosystems BigDye Terminator v3.1 Cycle Sequencing kit and Sanger sequenced at Edinburgh Genomics (University of Edinburgh, UK). Full-length ALVE insertions required eighteen sequencing reactions, using primers designed from the ALVE1 reference sequence (GenBank: AY013303.1) spaced every 500 bp (Table S6; Figure S2). Consensus sequences were built using the Geneious v7.0.4 [67] ‘Map to Reference’ tool, ALVE domains and SNPs were annotated, and LTR pairs were aligned by MUSCLE v3.8.31 [68]. ORFs were identified in each sequence by GLIMMER3 [69], transcription factor binding sites annotated by the EMBOSS v6.6.0 [70] tfscan tool, and the EMBOSS fuzznuc tool was used to identify the miR-155 AGCATTA target sequence in the ALVE *envelope* domain [71].

### High resolution optical mapping of the K locus

Whole blood samples were collected and stabilised in agarose plugs for one male individual from each of the HL WPR lines as well as one from slow feathering and fast feathering WLs. High molecular weight DNA was extracted at The Earlham Institute (UK) and analysed using the BioNano Irys platform with the Nt.BspQ1 restriction enzyme. Molecule object files were assembled and aligned to an in silico digest of the Galgal5 Z chromosome in IrysView v2.5.1 using the associated BioNano Knickers, Refaligner and Assembler software (release v5122; available: https://bionanogenomics.com/support/software-downloads/).

### Analysis of ALVE integration site distribution

ALVE integration sites were overlapped with the Ensembl v87 Galgal5 feature GTF and the locations of all RepBase [72] repeat classes (20181026 release). GC bias was inspected across each target site duplication and windows of 100 bp, 1kbp, 10kbp and 100kbp centred on the integration. Values were compared to the genome and chromosomal average, as well as simulations of an equal number of random integrations repeated one million times.

A matrix was constructed for the presence/absence of each ALVE within each analysed dataset, and used to build a hierarchical binary cluster tree based on Jaccard distances. A general linear model (GLM) was fitted to identify whether differences in sequencing library type (individual vs pool) or average genome coverage influenced ALVE identification by obsERVer when lines were grouped into five broad categories: white-egg layers, brown-egg layers, broilers, native breeds, and ‘wild’, including the RJF samples. GLM category groups are shown in Table S1.

## Results

### Detection of ALVE integrations in HL elite layer lines by obsERVer

We have developed obsERVer (https://github.com/andrewstephenmason/obsERVer), a focused bioinformatic pipeline for the detection of ALVE integrations in WGS datasets, which utilises popular, freely available tools for processing next generation sequencing data. obsERVer was initially used to identify twenty different ALVEs across eight elite layer lines from Hy-Line International, of which four ALVEs were novel to this study (Table 1). All detected ALVEs were present in the full genome alignment maps, and no other known ALVE sites (Table S3) were detected, including the two ALVEs of the reference genome (ALVE6/ALVE-JFevA and ALVE-JFevB) [50].

**Table 1:** ALVEs of the Hy-Line elite layer lines

Name	Location	TSD	Gene	Length	WL1	WL2	WL3	WL4	WL5	WPR1	WPR2	RIR
ALVEB5	1:10,637,460	GGTGGT		7530 (F)						AD	✓	✓
ALVE1	1:65,993,542	ACGGTT	SOX5 int1	7530 (F)	✓	✓	✓	✓	✓			
ALVE_ros001 (COTW55)	1:101,668,931	GTTGTG		7531 (F)								✓
ALVE_ros002 (COTW69)	1:158,775,708	ATAAGT		–								✓
ALVE_ros003 (SGT-24)	1:163,248,553	CCTACT		7528 (F)								✓
ALVE-TYR	1:187,921,213	ACACTG	TYR int4	7534 (F)						✓	✓	
ALVE-NSAC1	2:120,868,843	CCTGTT		4838 (P-E-3 L)						✓	✓	✓
ALVE_ros004	2:124,432,997	CTTGAC		7530 (F)						NSI	NSI	✓
ALVE_ros005 (New11)	2:142,480,536	TTGATA		280 (SL)						NSI		✓
ALVE-NSAC3	3:53,639,776	ATAAAA		–						✓	✓	
ALVE_ros006 (N4)	3:57,337,987	GGACTC		–								✓
ALVE15	3:70,384,294	GTTTAT	GRIK2 int16	280 (SL)	✓	✓			✓			
ALVE_ros007	4:59,843,015	AATAGA		1400 (E-3 L)								✓
ALVE_ros008 (BK-59)	4:62,680,158	CTGTAG		7529 (F)				✓				
ALVE_ros009	4:71,095,932	GTCCAG		–							✓	
ALVE9	6:33,153,441	CTCAAA	DOCK1 int35	5077 (P-E-3 L)			✓					
ALVE-NSAC7	9:11,714,130	CTTCTC		7531 (F)						✓	✓	
ALVE_ros010	9:11,871,576	TCGGAT		–						NSI		✓
ALVE3	20:10,309,347	AACCAC	HCK int6	5848 (F, RT-)		✓	✓	✓				AD
ALVE21	Z:10,681,671	GGGTAG		7529 (F)				✓		✓	✓	

Identified ALVEs are shown with Galgal5 location, target site duplication (TSD), overlap with annotated gene, sequence integrity, and presence in each of the eight analysed lines. Ambiguous prior names are shown. ALVE integrity is shown under length where: F = full, P = polymerase, E = envelope, 3 L = 3’LTR, RT- = missing reverse transcriptase, SL = solo LTR. Five ALVEs were not sequenced. ALVE detection: tick indicates detection by obsERVer; AD shows allelic dropout in the sequencing data, NSI shows that ALVE was present in the line, but not in the sequenced individuals.

In nineteen cases, manual inspection of the ALVE alignments in IGV was simple, with elevated coverage of the TSD and split reads supporting both the 5′ and 3′ ends of the ALVE integration. The novel ALVE_ros007, however, was initially identified by obsERVer as two separate sites 1939 bp apart due to a post-integration deletion of over 8 kbp, excising the absent genomic sequence (intergenic with no predicted regulatory or conserved regions) and over 80% of the ALVE integration (Figure S3). Manual inspection of obsERVer-identified ALVEs therefore remains crucial, particularly when detected sites are only supported by 5′ or 3′ split reads alone.

**Fig. 2:**
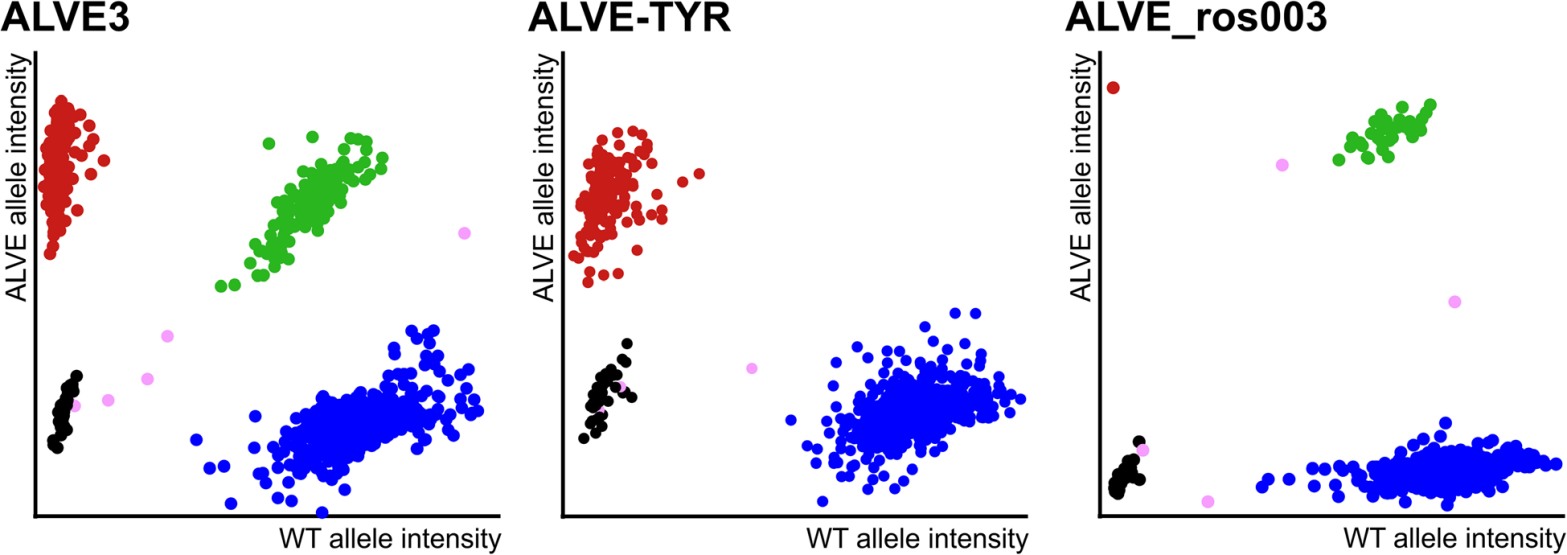
High-throughput genotyping of ALVE integrations by KASP assay. Genotype calls are based on relative intensity of the fluorescent tags conjugated to the wildtype and ALVE integration specific primers, with each dot representing a single chicken DNA sample. Red points (top left) represent individuals homozygous for the ALVE integration, blue points (bottom right) for individuals homozygous for the wildtype allele, and green points (top right) are heterozygotes. Black points are sample negative controls and pink points are ambiguous samples either due to their location outside genotype clusters, or their relative fluorescence positions at earlier cycle stages. Three plots are shown as examples, with all twenty in Fig. S4. ALVE3 shows high numbers of individuals in all three genotypes. ALVE-TYR is fixed in both WPR lines and absent in all WLs and the RIR (hence no heterozygotes). Homozygous individuals for ALVE_ros003 were rare, so genotype confidence was derived by pooling data from multiple generations (not shown)

We validated the bioinformatically-detected ALVEs by developing specific high-throughput KASP™ genotyping assays for each identified integration (Fig. 2; Figure S4; Table S4), and genotyped the original eighty males used for sequencing. Strikingly, obsERVer exhibited a 0% false discovery rate (FDR) as all bioinformatically identified ALVEs were subsequently detected by KASP assay in their appropriate lines. However, by KASP assay alone, ALVE3 was detected in a single RIR bird and ALVEB5 in a single WPR1 bird; neither of which were identified by obsERVer (Table 1). The full genome alignment files for each dataset showed no supportive read evidence for either integration, supporting the loss of both sites due to allelic dropout in the sequencing pools. Further genotyping of over 9000 males from multiple generations across the eight elite layer lines identified a further four ALVE occurrences within the WPRs (at frequencies < 0.1) which were not found in the smaller subset of sequenced birds (Table 1).

With a 0% FDR, obsERVer is a highly specific detection tool limited only by the experimental design of the sequencing project. The HL WGS datasets, limited by technology at the time, were from 10-individual pools and had low average coverage of 11-18X. Based on our modelling of integration detection, only ALVEs with a flock frequency of 0.3–0.5 had a > 90% probability of detection. At frequencies of 0.1 there was only a 28–45% chance of detecting that integration. Whilst this may support the use of target enrichment sequencing (such as [23]), WGS using individual libraries with high coverage is now commonplace, resulting in > 90% probability of detection with ALVE frequencies < 0.1 at 30x coverage. Rare ALVEs may still be missed due to population sampling, but target enrichment sequencing would not improve this.

### Characterisation of the obsERVer-identified ALVEs in the HL elite layer lines

Of the twenty identified ALVEs, eleven were shared between multiple lines (of which five were between multiple breeds; Table 1) giving a total of forty-two ALVE occurrences across the HL datasets. The white-egg layer WLs had fewest ALVEs with two to four loci per line, but these were often fixed or at high frequency within each flock. The three brown-egg layer lines had a greater number of ALVE loci (eight and nine in the WPRs; 11 in the RIR), typically found at flock frequencies < 0.2, likely reflecting the broader genetic background of these breeds [73]. All four novel ALVEs identified in this study were intergenic and found in brown-egg layers.

Five ALVEs were within introns (Table 1). Except for ALVE-TYR, where the integration in the final *TYR* (Tyrosinase) intron causes transcript truncation [44, 45], none of these has any reported effect on their containing gene. Interestingly, ALVE15, a solo LTR widespread in layers [21], is within the final intron of *GRIK2* (Glutamate Ionotropic Receptor Kainate Type Subunit 2) and Ensembl reports a *GRIK2* transcript which lacks the final exon, corresponding to the entire intracellular domain known to regulate channel dynamics in other glutamate receptor family members [74].

Fifteen of the twenty ALVEs were fully sequenced (Table 1; AF2). Of these, ALVE15 and ALVE_ros005 were solo LTRs, and ALVE9, ALVE-NSAC1 and ALVE_ros007 had varying 5′ truncations. The remaining ten were ‘intact’ elements with both 5′ and 3′ LTRs, although ALVE3 had no *reverse transcriptase* domain, matching the GenBank reference (AY013304.1). ALVE LTRs retained high identity (98.6% across all LTRs; 9/10 intact element LTR pairs had 100% identity; ALVEB5 5′ LTR had a single SNP at G262T) and contained intact TATA boxes, transcription start sites, and two binding sites for serum response factor (SRF). The high sequence similarity and integrity seen for the LTRs was not observed throughout the internal coding domains. Six of the ten ALVEs with a gag domain contained one or more mutations in the p10 or p27 that truncated any potential transcripts (Figure S5). Intact p27 was detected from ALVE1, ALVE3, ALVE21 (the only ALVE with the potential for total expression) and ALVE-TYR. Whilst ALVE1 is not normally expressed [33, 75, 76], the others have well characterised expression in both commercial and experimental lines [27, 44]. Sequence integrity was better across the envelope domain (Figure S6), with ten of the thirteen represented ALVEs containing unbroken reading frames with four to six non-synonymous changes. However, envelope expression may be inhibited by the intact miR-155 target site found in all ALVEs (position 5634–5640 relative to AY013303.1) [71]. No sequence was obtained for the remaining five ALVEs, however the original split reads suggest these elements had intact LTR pairs.

### ALVE21 and a K locus revertant

ALVE21 is an integration of great commercial interest as it is associated with the *K* locus mutation; a 180kbp tandem duplication on the Z chromosome which leads to gender-dependent, slowed feathering rate in day old-chicks [39, 42, 43]. The slow/fast feathering phenotype is used extensively in the production industry as a rapid non-invasive means of determining gender at hatch. Consequently, ALVE21 is often present in commercial flocks, even though it is structurally intact (as shown above) and retains the potential to produce retroviral proteins [29].

**Fig. 3:**
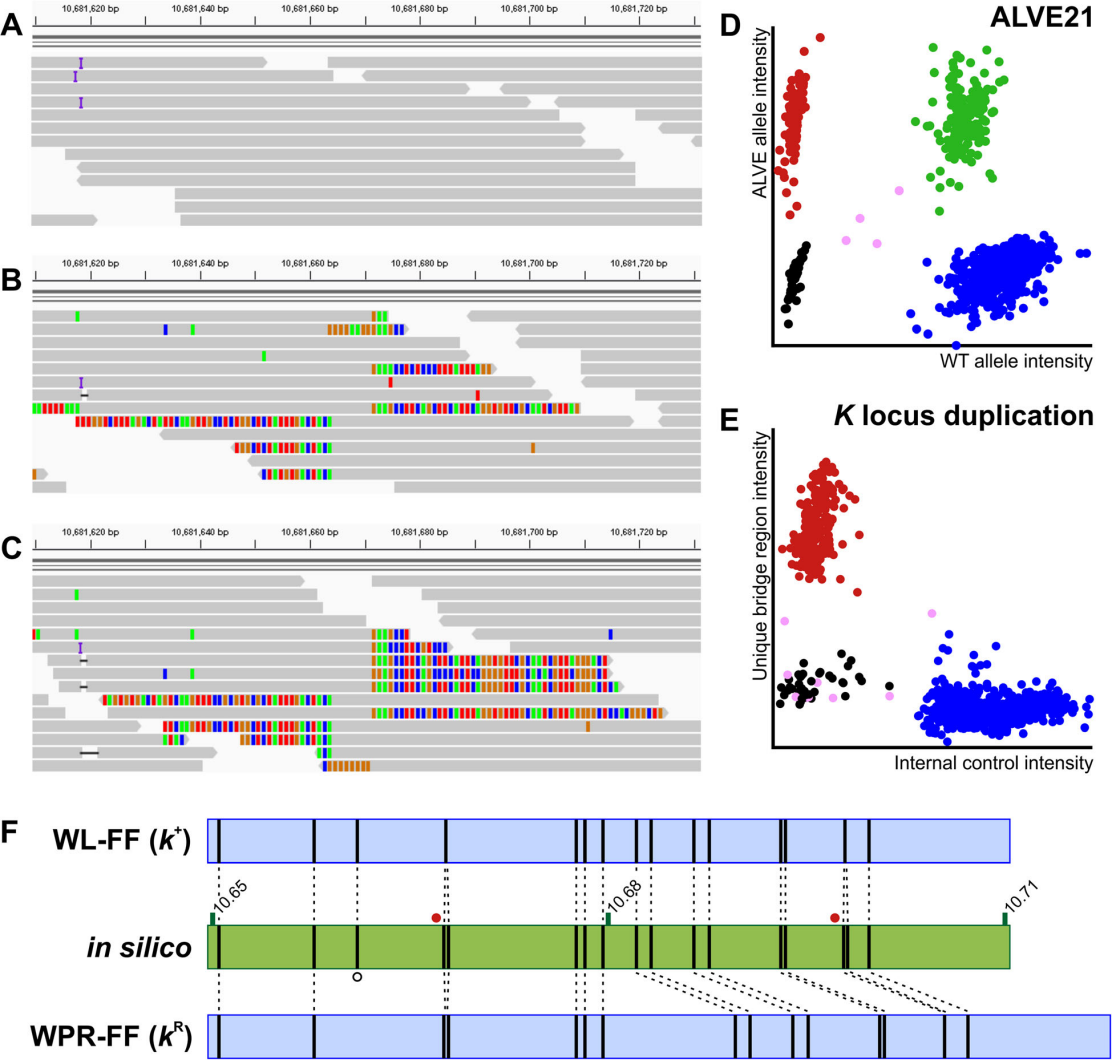
ALVE21 genotyping and identification of the WPR *K* locus revertant (*k*^R^). **a** IGV alignment view of the empty ALVE21 integration site in a FF WL. **b** IGV alignment view in the SF WPR. Base mismatches are coloured and show the split reads on either side of the TSD. Approximately 50% of reads align through the TSD, supporting the empty ALVE21 integration site in the tandem repeat of the *K* locus. **c** IGV alignment view in the FF WPR showing the ALVE21 integration, but with no reads aligning across the TSD. **d** ALVE21 KASP assay. All FF WPR individuals appear homozygous for ALVE21. All SF individuals appear heterozygous due to the empty site in the tandem repeat. **e** KASP assay for the unique bridging sequence between the two tandem repeats is only seen in the SF lines and not in any FF, including the FF WPR. Plot values corrected for representation of the internal control in all groups. **f** Optic maps generated across the *K* locus (coordinates show Mbp on the Z chromosome) using the Nt.BspQ1 restriction enzyme. The in silico shows predicted Nt.BspQ1 sites (vertical bars). Cases where predicted sites are very close (red circles) cannot be resolved beyond a single site. Predicted site dropout (open circle) may represent a mutation in that Nt.BspQ1 site. The WL-FF optic map matches the in silico exactly. The WPR-FF shows a ~ 7.5 kbp longer optic map representing the integrated ALVE21. Optic map figures were adapted from IrysView

ALVE21 was detected by obsERVer in both of the “slow feathering” (SF) HL lines (Fig. 3a-b), and by KASP assay (Fig. 3d) all SF birds appeared heterozygous, as ALVE21 is only found in one of the *K* locus tandem duplication sites (Figure S7). However, ALVE21 was also found in the wildtype, “fast feathering” (FF) WPR sister line, but exclusively in a homozygous state (Fig. 3c-d). This supports a phenotypic reversion (K^R^) by recombination in the FF WPR (as has been previously reported [77]), retaining the copy containing the ALVE21 integration (Figure S7). We validated this by designing a KASP assay to the unique bridging sequence between the tandem duplications. Congruently, both SF lines were homozygous for this sequence, but it was absent in all FF lines, including the phenotypic revertant (Fig. 3e). The ALVE21 integration in the FF WPR was also detected using BioNano high resolution optic mapping relative to a truly wildtype FF WL (Fig. 3f). Unfortunately, there was insufficient molecule resolution to fully describe the *K* locus in any SF individual or combined dataset (Table S7).

### Application of obsERVer to reveal broad ALVE diversity

Following validation of the twenty ALVE integration sites identified in the HL lines (with 0% FDR), obsERVer was used to identify ALVEs from fifty-seven diverse chicken WGS datasets (Table S1). These included experimental and heritage layer and broiler lines, further commercial layer lines, African and Asian native breeds, and wild-caught RJF from China, Java and Sumatra. When combined with the HL ALVEs, a total of 322 different ALVEs were identified by obsERVer in this study, of which 261 (81.1%) were novel (AF1). Neither of the two ALVEs of the reference genome were identified in any dataset, even the wild-caught RJF samples. All identified datasets contained at least one ALVE, except the ADOL Line 0 which had been selected to be ALVE free [37].

**Fig. 4:**
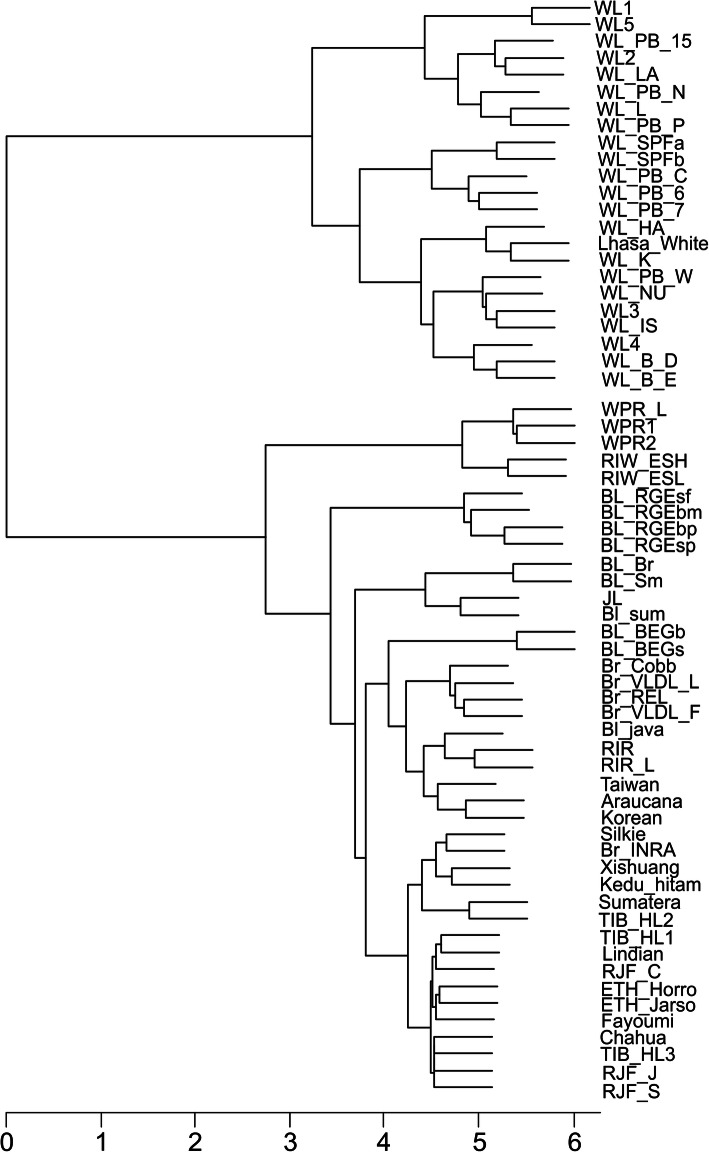
ALVEs as genetic markers. Cladogram constructed based on ALVE presence/absence data for all sixty-five analysed datasets (Table S1; AF1). WLs dominate and cluster tightly together in the top clade. Brown Leghorns cluster with the brown egg layer WPRs, RIWs and RIRs, as well as the heritage broiler datasets, reflecting the broader genetic diversity of these breeds. The non-commercial datasets are highly diverse

The WL samples had the fewest ALVEs (up to six per dataset) and formed a distinct clade in the dendrogram constructed using ALVE content (Fig. 4). Twelve of the nineteen ALVEs found in WLs had been identified in previous studies, and “typical” WL ALVEs were highly prevalent: ALVE1 (22/23), ALVE3 (14/23), ALVE9 (7/23) and ALVE15 (10/23). Brown egg layers, including WPR, RIR and RIW lines, had higher ALVE content (six to eleven) and the heritage broilers were higher again (thirteen to thirty), with many sites shared between these lines. Thirty-six of the eighty-three ALVEs found in commercially relevant breeds had been identified previously. Other, non-commercial WGS datasets were highly variable in their ALVE content, and also exhibited high lineage-specificity. Across the entire study, 260 ALVEs were specific to a single dataset (80.7%) with over 60% of these identified in the village chickens and wild-caught RJF.

It is unlikely these totals represent a comprehensive catalogue of ALVE content in these lines, particularly as some WGS datasets were derived from only a single bird, or a pool of 2–3 birds. Despite this, over 90.3% of the variation in ALVE content across the analysed datasets was due to line category (largely based on breed; Table S1): the only significant variable in the GLM (*P* < 10^− 4^). Genome coverage and derivation from an individual or pooled sequencing library were both non-significant terms.

### ALVE integration site distribution

Previous studies of exogenous ALV integration sites suggested a preference for open chromatin, particularly near protein-coding genes [78–80]. Under a model of random integration 51.8% of sites would fall within coding regions, however only 26.7% of the 322 ALVEs identified in this study were within genes; a significant depletion (*P* = 2.0 × 10^17^). However, 32.9% of ALVEs were within 10kbp of a gene, compared to just 4.1% in the random integration model (*P* = 3.1 × 10^− 147^). Furthermore, whilst there was no observable GC bias at any window size across the integration sites, the 6 bp TSDs were significantly more GC rich than expected (49.9% GC compared to genome mean of 42.4%; t = 4.66; *P* = 3.8 × 10^− 6^). Taken together, these results support the previous ALV integration studies [78, 79], with depletion within genes likely due to post-integration selection on the host genome.

ALVE_ros012 (found only in the RJF-J dataset; Table S1) was the only ALVE found within an exon (exon 8/11 of carboxypeptidase A5 precursor; *CPA5*), and would likely cause a truncation of 170 amino acids (40.6%). The impact of this truncation on the host is unknown, but may be mediated by multiple *CPA5* paralogues, including the neighbouring *CPA1* and *CPA2*. Intronic integrations (25.8% of all identified sites) may also elicit effects on the host organism by causing truncations or exon skipping, as with ALVE-TYR [44].

A total of eighteen ALVEs (5.6%) were found to have integrated within assembled Chicken Repeat 1 (CR1) elements within the genome (AF1). These included thirteen novel integrations (including ALVE_ros009 identified in the HL elite layer lines), as well as the previously described ALVE12, ALVE16, ALVEB10, ALVE_NSAC2 and ALVE_NSAC5. The observed number of ALVEs within assembled repetitive elements closely matched that of the random integration model (5.7%; *P* = 1.00).

## Discussion

### obsERVer enables detection of specific retroviral integrations from WGS data

High-throughput sequencing technologies have facilitated the description of many genomic features, although repetitive elements remain relatively understudied. Whilst repeat element-targeted sequencing technologies (such as [23]) seem appealing, the generated data are not typically applicable to other research questions, with data repurposing usually a major strength of sequencing projects. Here, we have described obsERVer, a pipeline developed for the identification of specific, user-determined retroviral integrations in existing WGS data, and then applied it to the identification of ALVE integrations in sixty-five chicken datasets, describing 322 ALVEs, of which 261 were novel (including 6 within commercial lines).

Development of diagnostic assays to 20 ALVEs identified across eight elite layer lines revealed a 0% FDR for obsERVer in detecting ALVE integrations, making it a highly precise method for identifying integration sites. It is unlikely that the ALVEs identified in this study represent a complete annotation of all integration sites within the WGS examined for this study. Integrations within difficult to sequence, or poorly assembled regions of the genome will not be detected, currently limiting any identification on many of the microchromosomes, the W chromosome, or near the centromeres and telomeres. For example, ALVE6 is common in commercial lines [21] but was not found in this study, likely due to its location near the chromosome 1 p arm telomere and its incomplete assembly in Galgal5. Further reference genome improvements will aid ALVE identification; recently shown specifically with ALVE6 [50].

Beyond the genome itself, the sequencing strategy also impacts obsERVer annotation completeness. As we saw in the HL data, rare ALVEs in a flock may be missed due to the specific individuals chosen for sequencing, and from allelic dropout from pooled sequencing libraries. With higher coverage and individual sequencing libraries now typical, allelic dropout is of less concern in future projects, however researchers should consider the minimum number of individuals needed to identify rare integrations. Target-enriched sequencing from multiple pooled-individual libraries (with high coverage) might be a more cost-effective way to ensure identification of all ALVEs in a population if the sole purpose of the investigators is to design genotyping assays to those integrations.

### The biological impact of ALVEs in chicken populations

A total of 322 different ALVE integrations were identified in this study. We confirmed previous work showing that commercial layers had fewer ALVEs than broilers [21, 81], and our novel assessment of non-commercial populations suggests that intensive poultry selection has successfully limited ALVE abundance within flocks. The greater number of highly lineage-specific ALVEs in non-commercial and RJF populations may suggest a high ancestral diversity of ALVEs before domestication [73, 82, 83], and would be consistent with recurrent infection and a role for ALVEs in ERV derived immunity (EDI) against exogenous ALV [84, 85]. Moreover, the RJF reference genome does not appear to be representative of observed ALVE diversity as it contains only two ALVEs [49, 50, 86]. A broader analysis of non-commercial and RJF datasets is needed to assess the role of ALVEs in wild populations, particularly as the structural integrity of each integration cannot be unambiguously determined from short read sequencing data alone.

The lower ALVE number in commercial lines is likely due to a combination of detrimental associations with productivity traits, relatively small effective population sizes, and the narrow genetic background of some breeds; factors which are less prominent in broilers compared with layers [73]. Flocks have also been subjected to decades of selection against the ALV-specific p27 antigen, and degradation of this region was seen in six of the ten HL ALVEs with an intact *gag* domain. However, with breeding programmes focused on multiple traits, and the close association of some ALVEs with desirable phenotypes, many ALVEs in commercial lines are found at very high frequencies, or have become fixed. Traditional selective breeding methods could gradually reduce ALVE allele frequencies, but fixed ALVEs, such as ALVE21 and ALVE-TYR in both HL WPRs, could only be removed by out crossing, which would likely create varied, undesirable production phenotypes. The CRISPR/Cas9 system was recently used to eradicate porcine ERVs from the pig genome [87], and could be an approach applied to commercial poultry to remove ALVEs such as ALVE21 whilst maintaining the associated slow feathering phenotype. Furthermore, accurate integration site identification by obsERVer facilitates highly specific genome editing for ALVE removal. Use of the high-throughput diagnostic KASP assays developed in this study have begun to identify phenotypic effects of segregating ALVEs in the HL flocks [88], and will identify priorities for future breeding programmes.

## Conclusions

We have developed and utilised the obsERVer pipeline to identify 322 ALVE integration sites across sixty-five chicken WGS datasets without the need for additional targeted sequencing. Further work is needed to elucidate the biological impact, if any, of these ALVEs on exogenous ALV infection modulation, and on productivity traits. Development of high-throughput diagnostic assays will enable better management of ALVEs in commercial stock and may lead to their eventual eradication in these lines. Beyond ALVEs, obsERVer can be applied to the identification of any retroviral integration in any species.

## Supplementary information

**Additional file 1: AF1.** Presence/Absence matrix for all identified ALVEs. This file gives the location, names, previous names and any gene or repeat element overlaps for each of the identified ALVEs as well as their presence (1) or absence (0) within each analysed dataset. Dataset names are given as the codes listed in Table S1.

**Additional file 2: AF2.** Hy-Line ALVE sequences. This file contains all fifteen successfully sequenced ALVEs from the eight Hy-Line elite layer lines. FASTA headers include sequence genomic orientation, observed length and new GenBank accession numbers where applicable.

**Additional file 3: AF3.** Supplementary figures. This file includes seven additional figures which support the manuscript. These are referred to in the text as Fig. S1 etc. Full titles and legends are given for each figure.

**Additional file 4: AF4.** Supplementary tables. This file includes seven additional tables which support the manuscript, including lists of primers. These are referred to in the text as Table S1 etc. Full titles and legends are given for each table.

## Data Availability

The obsERVer bioinformatics pipeline is freely available on GitHub (https://github.com/andrewstephenmason/obsERVer). Accession values for publicly available WGS data have been indicated, or the relevant publication for non-public data (Table S1). Eleven previously unpublished ALVE sequences derived in this study have been uploaded to GenBank with accession numbers indicated in AF2.

## Abbreviations

ADOL: Avian Disease and Oncology Laboratory
ALV: Avian Leukosis Virus
ALVE: Avian Leukosis Virus subgroup E
ART-CH: Avian retrotransposon of chickens
BL: Brown Leghorn
bp: Base pairs
CR1: Chicken Repeat 1
EAV: Endogenous avian virus
EDI: ERV-derived immunity
ERV: Endogenous retrovirus
FDR: False discovery rate
FF: Fast feathering
GLM: General linear model
HL: Hy-Line
KASP: Kompetitive Allele-Specific PCR
kbp: Kilobase pairs
LTR: Long terminal repeat
MDV: Marek’s Disease virus
NGS: Next generation sequencing
PCR: Polymerase chain reaction
RFLP: Restriction fragment length polymorphism
RIR: Rhode Island Red
RIW: Rhode Island White
RJF: Red junglefowl
SF: Slow feathering
SNP: Single nucleotide polymorphism
SRF: Serum response factor
T_m_: Melting temperature
TSD: Target site duplication
UTR: Untranscribed region
WGS: Whole genome sequencing
WL: White Leghorn
WPR: White Plymouth Rock
