# Supplementary material for: Identification and characterisation of endogenous Avian Leukosis Virus subgroup E (ALVE) insertions in chicken whole genome sequencing data
Source: Mob DNA. 2020 Jun 30;11:22. doi: 10.1186/s13100-020-00216-w (PMC7325683; doi:10.1186/s13100-020-00216-w)

# Figure S1: obsERVer pipeline workflow

obsERVer consists of seven scripts shown here as S1-7. Assembled endogenous alpharetroviruses are first identified in the reference genome (S1) and a pseudochromosome is constructed using reference ALVE sequences (S2). WGS reads are mapped to the “pseudochromosome” (S3) and mapped reads and their mates are subtracted (S4) and mapped to the reference genome (S5). Putative sites are derived by filtering out reads mapped to assembled endogenous alpharetroviral locations (S6), and output files generated ready for manual validation in IGV (S7).

All scripts are available on GitHub: https://github.com/andrewstephenmason/obsERVer


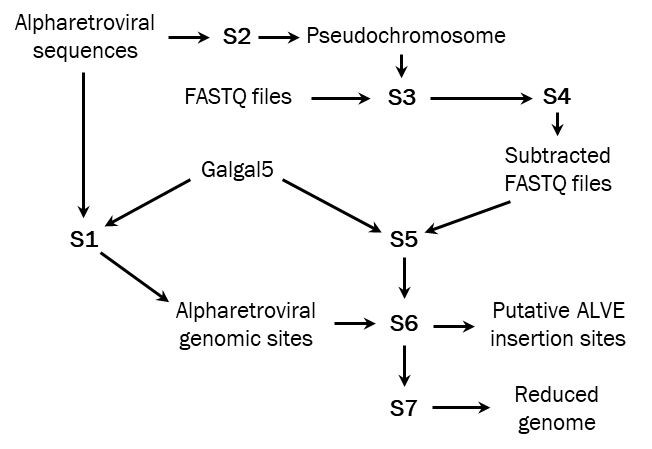


# Figure S2: ALVE generic sequencing primer locations.

Primers were designed using the ALVE1 reference sequence (GenBank: AY013303.1). The alternating black and grey backbone represents 500bp stretches of the 7.5kb element. The triangles show the primer locations and directions, with red sequencing the forward strand, and blue the complementary strand. The 5’-3’ order of the primers is the same as in Table S6. Domains: matrix (p19 and p10), capsid (p27), nucleocapsid (p12), protease (PRO), reverse transcriptase (RT), integrase (INT), surface (gp85), and transmembrane (gp37).


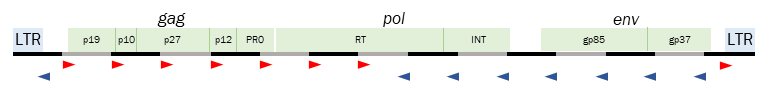


# Figure S3: The ALVE_ros007 genomic region.

ALVE_ros007 was initially identified as two separate integration sites in the HL RIR dataset. However, the 5’ site had only 3’ end split reads, and vice versa. The IGV plot (A) shows the RIR full genome alignment map along this region of chromosome 4 (from 59.843 – 59.845 Mbp) below the gradated scale, and the reads above show the reads with ALVE homology extracted by obsERVer. Split reads at the 5’ end of the locus have homology to the 3’ ALVE LTR, and reads at the 3’ end have homology to the ALVE *envelope* domain. This suggests the true integration site is at 4: 59,843,015 (the 5’ end) and there has been a deletion of 8,064 bp (6,123 bp of the ALVE integration and 1,941 bp of the ‘wildtype’ genome). A schematic for this mode of integration and deletion is shown in B: 1) wildtype sequence with future ALVE_ros007 integration site marked by vertical black line; 2) ALVE integrates; 3) dotted box indicates the extent of the 8,064 bp deletion; and 4) the resulting shorter sequence. In the HL RIR line all individuals had either the wildtype or combined ALVE and deletion alleles.


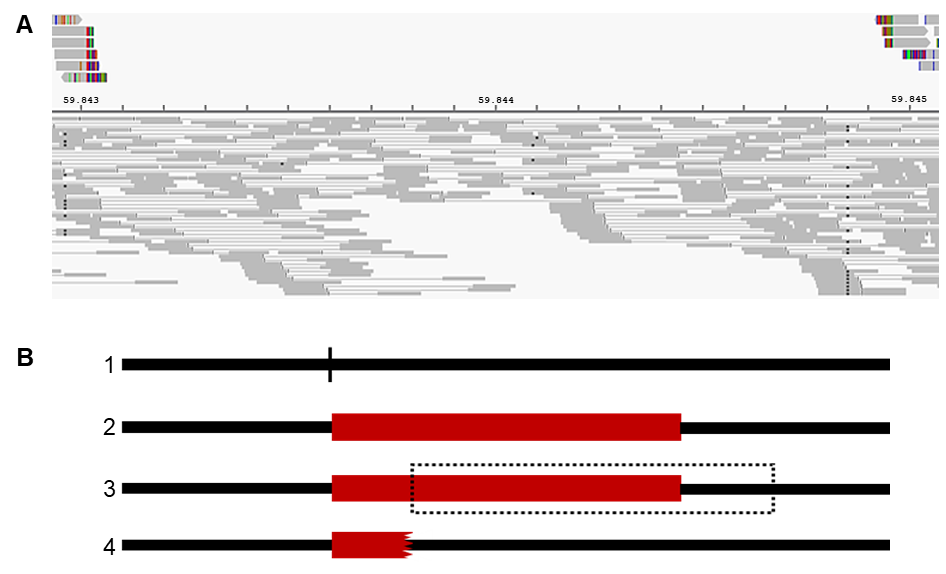


# Figure S4: KASP assay results for ALVEs identified in the Hy-Line elite layer lines.

Each subplot is an independent assay with the specific ALVE indicated. The y axis represents relative intensity of the ALVE-specific fluorescently labelled primer, and the x axis represents the same for the wildtype allele. Therefore, red data points (top left) are homozygous for the ALVE integration, green (top right) are heterozygotes, and blue (bottom right) are homozygous for the wildtype allele. Pink data points were ambiguous either due to their location outside called genotype clusters, or the path taken during cycling. This could be assessed as data points were genotyped multiple times during PCR amplification and the best cycle chosen for the images. Black data points are control negative samples. These plots represent a single generation of all males across all eight sequenced flocks. For assays where the insert allele was rare (*e.g.* ALVE_ros003 or ALVE_ros005), confident genotype clusters were called using multiple generations.


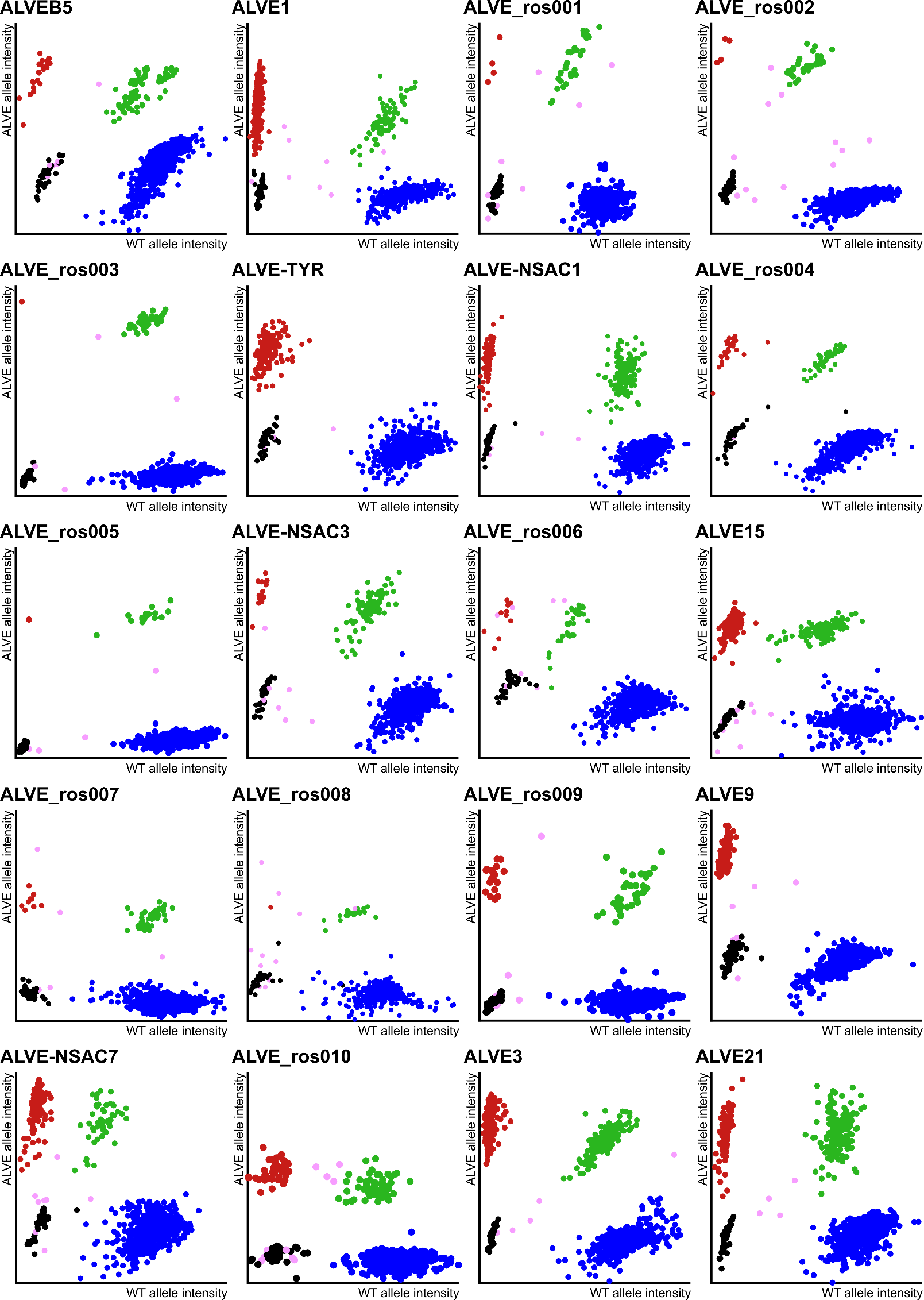


# Figure S5: Schematic of the gag-pol ORFs detected in each of the twelve sequenced ALVEs containing gag or pol sequence.

Each blue bar represents a continuous ORF with dotted lines showing non-continuous ORFs. The solid line connecting the ORF blocks of ALVE3 shows contiguity, but the absence of PRO and RT. Abbreviated pol domains are: PRO = *protease*, RT = *reverse transcriptase*, INT = *integrase*.


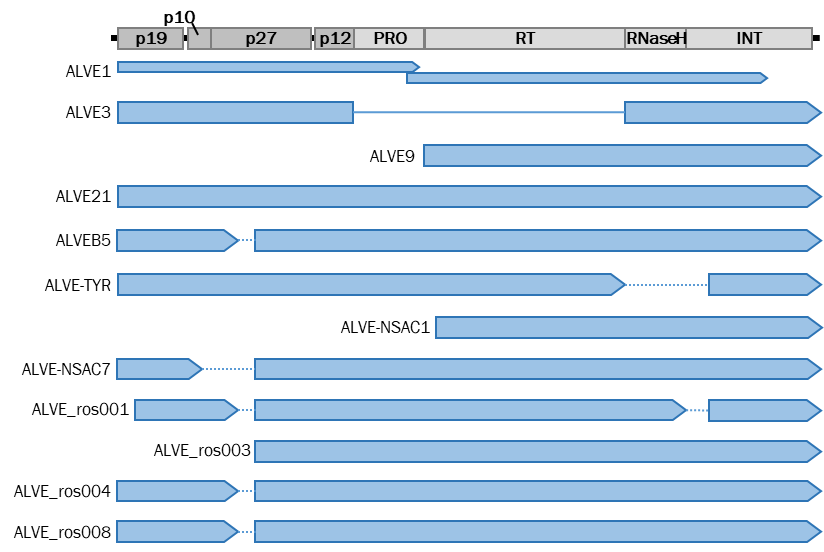


# Figure S6: Schematic of the *envelope* ORFs identified in each of the thirteen sequenced ALVEs which contained the *envelope* domain.

*Envelope* schematic showing surface (SU) and transmembrane (TM) domains and the open reading frames identified in the three ALVEs with non-intact *envelope* ORFs compared to the intact ORF. The miR-155 binding domain (AGCATTA) is 208bp into the 978bp SU domain.


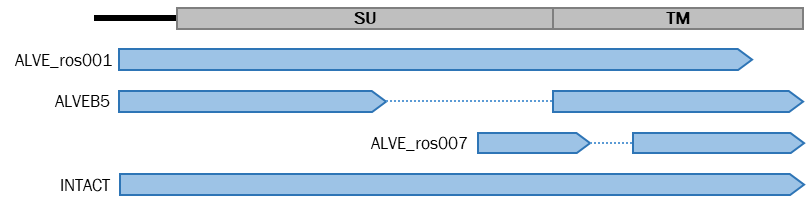


# Figure S7: Mechanism and outcomes for *K* allele reversions.

The tandem repeats of the K allele retain greater than 99 % homology so will readily recombine producing phenotypic revertants. With respect to ALVE21, there are two possible revertant genotypes, with (k^R^) or without (k^+^; reversion to ‘true’ wildtype) the insertion. These genotypes will depend on where recombination occurs.


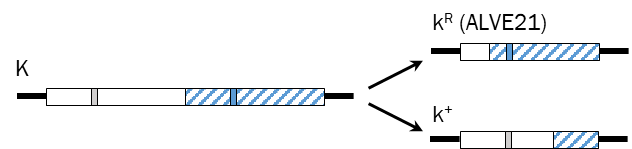

Supplement: Supplementary file 3 — Additional file 3: AF3. Supplementary figures. This file includes seven additional figures which support the manuscript. These are referred to in the text as Fig. S1 etc. Full titles and legends are given for each figure. [file 13100_2020_216_MOESM3_ESM.docx]
