# Supplementary material for: Identification and characterisation of endogenous Avian Leukosis Virus subgroup E (ALVE) insertions in chicken whole genome sequencing data
Source: Mob DNA. 2020 Jun 30;11:22. doi: 10.1186/s13100-020-00216-w (PMC7325683; doi:10.1186/s13100-020-00216-w)
Supplement: Supplementary file 4 — Additional file 4: AF4. Supplementary tables. This file includes seven additional tables which support the manuscript, including lists of primers. These are referred to in the text as Table S1 etc. Full titles and legends are given for each table. [file 13100_2020_216_MOESM4_ESM.docx]

# Table S1: WGS datasets analysed for this study.

The datasets and the lines they contain are listed. Each dataset has in brackets a reference or contact name. Each line was given a coded name which is either a shortening of the full line name or includes the breed, where BL = Brown Leghorn, Br = broiler, RIR = Rhode Island Red, RIW = Rhode Island White, RJF = red jungle fowl, WL = White Leghorn, and WPR = White Plymouth Rock. The RJF abbreviations include their countries (C = China, J = Java, S = Sumatra) and the Ethiopian village birds are abbreviated with the international code for Ethiopia (ETH). The Tibetan highland breeds are abbreviated as TIB-HL. The library type is shown for each line, showing either the number of individuals used in each pool, or the number of individual (indiv) sequencing libraries available. All datasets were Illumina 101bp paired end. The final column assigns each line a group number (1-5) for GLM analysis. Group 1 is commercial white egg layers, group 2 is commercial brown egg layers, group 3 is broilers, group 4 is generalist and ‘native’ breeds, and group 5 is RJFs and ‘village’ chickens.

| Dataset | Line | Code | Library type | Group |
| --- | --- | --- | --- | --- |
| Arkansas vitiligo model (PRJNA256208) | Brown | BL-Br | Pool (10) | 1 |
|  | Smyth | BL-Sm | Pool (10) | 1 |
| Cobb heritage broiler (PRJEB15276) | - | Br-Cobb | Indiv (20) | 3 |
| Commercial 1995 broiler (Douglas Rhoads, University of Arkansas) | - | Br-REL | 8 x Pool (10) | 3 |
| Egg shell strength (PRJNA231017) | High | RIW-ESH | Pool (8) | 2 |
|  | Low | RIW-ESL | Pool (8) | 2 |
| Ethiopian village birds (Olivier Hanotte, University of Nottingham) | Horro | ETH-Horro | Indiv (6) | 5 |
|  | Jarso | ETH-Jarso | Indiv (5) | 5 |
| Fat-Lean broilers (PRJEB15288) | Fat | Br-VLDL-F | Indiv (4) | 3 |
|  | Lean | Br-VLDL-L | Indiv (4) | 3 |
| High and Low antibody (Chris Ashwell, North Carolina State) | High | WL-HA | Pool (16) | 1 |
|  | Low | WL-La | Pool (16) | 1 |
| Hy-Line elite layer lines  (Janet Fulton, Hy-Line International) | WL  WL  WL  WL  WL  WPR  WPR  RIR | WL1  WL2  WL3  WL4  WL5  WPR1  WPR2  RIR | Pool (10)  Pool (10)  Pool (10)  Pool (10)  Pool (10)  Pool (10)  Pool (10)  Pool (10) | 1  1  1  1  1  2  2  2 |
| INRA high/low fat cross (PRJNA247952) | - | Br-INRA | Indiv (16) | 3 |
| Indonesian natives (DDBJ DRA003951) | Black java | Bl-java | Pool (10) | 4 |
|  | Black sumatra | Bl-sum | Pool (10) | 4 |
|  | Kedu Hitam | Kedu hitam | Pool (10) | 4 |
|  | Sumatera | Sumatera | Pool (5) | 4 |
|  | Java RJF | RJF-J | Pool (2) | 5 |
|  | Sumatra RJF | RJF-S | Pool (3) | 5 |
|  | WL | WL-NU | Indiv (1) | 1 |
| Iowa State (Sue Lamont,  Iowa State) | Fayoumi | Fayoumi | Pool (16) | 4 |
|  | Leghorn | WL-IS | Pool (16) | 1 |
| Korean domestic (PRJNA291174) | Araucana | Araucana | Indiv (3) | 4 |
|  | Korean | Korean | Indiv (3) | 4 |
|  | WL | WL-K | Indiv (3) | 1 |
| Lohmann layers (Rudolf Preisinger, Lohmann) | RIR | RIR-L | Indiv (25) | 2 |
|  | WL | WL-L | Indiv (25) | 1 |
|  | WL | WL-Lp | Pool (10) | 1 |
|  | WPR | WPR-L | Pool (10) | 2 |
| Pirbright inbred lines (Dave Burt, The Roslin Institute) | 15 | WL-PB-15 | Pool (10) | 1 |
|  | 6 | WL-PB-6 | Pool (10) | 1 |
|  | 7 | WL-PB-7 | Pool (10) | 1 |
|  | C | WL-PB-C | Pool (10) | 1 |
|  | N | WL-PB-N | Pool (10) | 1 |
|  | P | WL-PB-P | Pool (10) | 1 |
|  | Wellcome | WL-PB-W | Pool (10) | 1 |
|  | Zero | WL-PB-Z | Pool (10) | 1 |
| Roslin experimental blind (Paul Hocking, The Roslin Institute) | BEG blind | BL-BEGb | Pool (10) | 1 |
|  | BEG sighted | BL-BEGs | Pool (10) | 1 |
|  | RGE blind | BL-RGEbm | Indiv (1) | 1 |
|  | RGE blind | BL-RGEbp | Pool (10) | 1 |
|  | RGE sighted | BL-RGEsf | Indiv (1) | 1 |
|  | RGE sighted | BL-RGEsp | Pool (10) | 1 |
| SPF commercials (Marc Eloit, Pasteur Institute) | A | WL-SPFa | Pool (14) | 1 |
|  | B | WL-SPFb | Pool (11) | 1 |
| Taiwanese domestic (PRJNA202483) | Silkie | Silkie | Indiv (1) | 4 |
|  | Taiwan Country | Taiwan | Indiv (1) | 4 |
| Tibetan highland/lowland (PRJNA309581) | Chahua | Chahua | Indiv (1) | 4 |
|  | Highland1 | TIB-HL1 | Indiv (1) | 4 |
|  | Highland2 | TIB-HL2 | Indiv (1) | 4 |
|  | Highland3 | TIB-HL3 | Indiv (1) | 4 |
|  | Lhasa White | Lhasa white | Indiv (1) | 1 |
|  | Lindian | Lindian | Indiv (1) | 3 |
|  | WL | WL-B-D | Indiv (1) | 1 |
|  | WL | WL-B-E | Indiv (1) | 1 |
| Tibetan fighting (PRJNA241474) | RJF | RJF-C | Indiv (6) | 5 |
|  | Xishuangbanna | Xishuang | Indiv (8) | 4 |

# Table S2: Reference alpharetroviral sequences used for identification of assembled sites in the Gallus_gallus5.0 genome assembly and creation of the pseudochromosome.

Names and accession numbers of alpharetroviral sequences used in the project. All sequences were used to identify assembled alpharetroviral sites within the reference genome, and those marked with ticks were used to create the alpharetroviral pseudochromosome. RSV was excluded from the pseudochromosome to remove *v-SRC* and the evJ/ALV-J sequences were excluded due to the presence of the EAV-HP homologous *envelope* domain.

| Sequence name | GenBank accession | Pseudochromosome? |
| --- | --- | --- |
| ALV-A | M37980.1 | ✓ |
| ALVE-B9 | KC610515.1 | ✓ |
| ALVE-B10 | KC610516.1 | ✓ |
| ALVE-B11 | KC610517.1 | ✓ |
| ALVE-NSAC-1 | FJ793550.1 | ✓ |
| ALV-J | Z46390.1 |  |
| ALV-J | KP284572.1 |  |
| ALV-LR9 | AY350569.1 | ✓ |
| ART | L25262.1 |  |
| ART | L25261.1 |  |
| EAV-0 | AM418554.1 |  |
| EAV-0-E51 | AM418555.1 |  |
| EAV-51 | AM418553.1 |  |
| EAV-E33 | AM418557.1 |  |
| EAV-E51 | AM418556.1 |  |
| EAV-HP | NC_5947.1 |  |
| EAV-HP | AJ238124.1 |  |
| EAV-HP | AJ238125.1 |  |
| EAV-HP | AJ623289.1 |  |
| ev1 | AY013303.1 | ✓ |
| ev3 | AY013304.1 | ✓ |
| ev6-env | AY013305.1 | ✓ |
| ev21 | Y12574.1 |  |
| ev-C11 | DQ500007.1 | ✓ |
| evJ | AF125527.1 |  |
| IC10 | X13744.1 |  |
| rav0-5prime | AH002471.1 |  |
| RAV-0-env | AF257657.1 |  |
| RAV-0-gag | M73497.1 |  |
| RSV | AF033808.1 |  |
| SE21Q1B-gag | M73496.1 |  |

# Table S3: ALVEs with previously published insertion sites, target site duplications (TSDs) or diagnostic assays which could be manually annotated across the Galgal5 assembly.

Suggested “common” or previously well characterised ALVEs with available data were used to manually inspect other regions of the Galgal5 assembly for ALVE integrations which were not detected by obsERVer. This list was supplemented by sites kindly shared by Professor Bernhard Benkel, as acknowledged. These sites are not detailed below. Assembly locations represent the coordinate position after the sixth base of the TSD.

| Name | Galgal5 location | TSD |
| --- | --- | --- |
| ALVE6 | 1: 576 | GGCGCT |
| ALVE-JFevB | 1: 32,603,304 | GGCTTG |
| ALVE2 | 1: 36,872,528 | GAGGGG |
| ALVE16 | 1: 67,449,967 | CATGGC |
| ALVE12 | 1: 122,259,940 | GTGTTG |
| ALVE-NSAC2 | 1: 146,108,486 | GGGTCC |
| ALVEB11 | 2: 62,587,235 | AGAGGA |
| ALVEB2 | 2: 95,058,383 | GACCAT |
| ALVE-NSAC5 | 3: 73,338,411 | GGCTGA |
| ALVEB10 | 4: 27,829,406 | GCATTC |
| ALVEB4 | 4: 88,793,982 | ATGTTT |
| ALVEB9 | 5: 12,126,222 | GGGGAC |
| ALVEB1 | 5: 23,238,400 | GTTATT |
| ALVE-NSAC6 | 5: 57,261,506 | AAAACT |
| ALVE4 | 6: 33,827,722 | GCTGCC |
| ALVEB3 | 7: 20,479,059 | GTAGTC |
| ALVE-NSAC4 | 12: 17,625,255 | CCTGGG |
| ALVEB6 | 14: 9,367,708 | GTGTCT |
| ALVEB8 | 20: 1,468,850 | GACTAC |
| ALVE7 | Z: 14,471,852 | ACCCTC |

# Table S4: Primers developed for the diagnostic Hy-Line-based KASP assays.

There are four primers for each assay (Figure 1). The first pair is for the ‘no insert’ genotype and the second pair is for the insert. Primers 1 and 3 for each assay have the fluorescent tags (sequences not shown, available from LGC). Base ambiguities are shown using the IUPAC codes. The K-duplication assay uses an internal control commonly used by Hy-Line (first primer pair), so individuals with the duplication appears heterozygous unless corrected.

| ALVE | KASP primers |
| --- | --- |
| ALVEB5 | 5'-AATAAACAATTCTCAGCTTAACCACCC  5'-GAATGTTTAAGTCYGTGGATCTAATGA  5'-AAAATAAACAATTCTCAGCTTAACCACCT  5'-TTGCGAACACCTRAATGAAGCAGAA |
| ALVE1 | 5'-AAATTGACTTTAATATCTGTCAACGGTTC  5'-CCAAGCAAGTAGCCCAAAACACAGTA  5'-AAATTGACTTTAATATCTGTCAACGGTTG  5'-GAACACCTGAATGAAGCAGAAGGCTT |
| ALVE-ros001 | 5'-TCATACACATGTTGTGYTCCCTG  5'-ATGTCCTGACTATGATCTGGCAGCT  5'-TTCATACACATGTTGTGTGAAGCCTT  5'-TAACGATTGCGAACACCTGAATG |
| ALVE-ros002 | 5'-TTCTGTGATTTAGTGATTCTATGATAAGTC  5'-CTACAATTCTGTGATTCTGTGATTCTG  5'-ATTCTGTGATTTAGTGATTCTATGATAAGTG  5'-CAAGTTGCCTCTGGCTCTATTTGACTA |
| ALVE-ros003 | 5’-TAGATCCTGATATCTTCATCCCTATCA  5’-ATTTATACAAATGTATGTGGTGAGAATGAT  5’-TAGATCCTGATATCTTCATCCCTATCT  5’-GAGTTGCCTCTGGCTCTATTTGACTA |
| ALVE-TYR | 5'-TTTCACTCTGAGCCTTCCAGTGTTA  5'-TGCAGTACCAGTGTATACAGATTGTGTAA  5'-TTCACTCTGAGCCTTCCAGTGTTG  5'-GAACACCTGAATGAAGCTGAAGGCTT |
| ALVE-NSAC1 | 5'-GTAAGCCTGGAGATGTCCTGTTC  5'-CAATACACAAGACTGAAAGCAGTCCAT  5'-GTAAGCCTGGAGATGTCCTGTTT  5'-GAACACCTGAATGAAGCTGAAGGCTT |
| ALVE-ros004 | 5'-CTCTTGCTTAATGTTTTGTTATCTTGACC  5'-TGGCAAATCGTTTCTGAGTCCAATTAGAT  5'-TCTCTTGCTTAATGTTTTGTTATCTTGACT  5'-CAAGTTGCCTCTGGCTCTATTTGACTA |
| ALVE-ros005 | 5’-CTAAATATGTCTTTTTTGTCTCCTTGATAC  5’-GGGTAAACAATAGCACTGCTCCTTAT  5’-ATTCTAAATATGTCTTTTTTGTCTCCTTGATAT  5’-TAGCGATTGCGAACACCTGAATGAA |
| ALVE-NSAC3 | 5'-AAACAGCTGATGGTATATCTTTTCATAAAATA  5'-CATTTCCATACACATCACAGAGATGAAATT  5'-ACAGCTGATGGTATATCTTTTCATAAAATG  5'-GAACACCTGAATGAAGCAGAAGGCTT |
| ALVE-ros006 | 5'-ATATTCAGACACAGGACTCC  5'-AGCCAGTCTGTATCTTCTGT  5'-CATATTCAGACACAGGACTCT  5'-TCTGGCTCGATTTGACTAC |
| ALVE15 | 5'-TAGAATATATTTACAAAAATCTCCATRTTTATG  5'-CTTTGAGGATCTACTTGATGAAAACATGTT  5'-TAGAATATATTTACAAAAATCTCCATRTTTATT  5'-TAACGATTGCGAACACCTGAACGAA |
| ALVE-ros007 | 5'-TCATAACAATGGAGATGTGGGAATAGATA  5'-TTGATCCAAGGCAGGTAGTATTATCTGTT  5'-TTCATAACAATGGAGATGTGGGAATAGATT  5'-CGTTGAGTCCCTAACGATTGCGAA |
| ALVE-ros008 | 5'-TATGATAAGAATTTTCTGTAGG  5'-AATATCTGAGACAGAGAATAAA  5'-CTATGATAAGAATTTTCTGTAGT  5'-AGACTATTCAAGTTGCCTCT |
| ALVE-ros009 | 5’-GGGTTCATGCTGTGTCCAGTG  5’-GAACACCTGAATGAAGCAGAAGGCTT  5’-GGGTTCATGCTGTGTCCAGTC  5’-AGATTTGCAGAGGGTGCACTCCAT |
| ALVE9 | 5'-TCCCTCTTGAGTCTCAAAAGAGTTC  5'-GAAAGCCTGTGTATTATTAAAGGCCC  5'-ATTCCCTCTTGAGTCTCAAATGAAGCC  5'-TCCCTAACGATTGCGAACACCTGAA |
| ALVE-NSAC7 | 5'-TGGATGAACAAGTTCACTTCTCTAAGA  5'-ACCAGACTGCATGTGTGTTAGCTTAACA  5'-TTGGATGAACAAGTTCACTTCTCTGAAGC  5'-ACGATTGCGAACACCTGAATGAAGCA |
| ALVE-ros010 | 5'-CTTATTTAGGAGAAATGCAAATGTAGGCTA  5'-TTTGTGTAATACCCCATTAGGGGCATAT  5'-ATTTAGGAGAAATGCAAATGTAGGCTG  5'-AAGTTGCCTCTGGCTCTATTTGACTA |
| ALVE3 | 5'-GCTCCACGGTCCGTGGTTG  5'-TGCAGTAATGGTGTTTGACACCAATTGAT  5'-GGCTCCACGGTCCGTGGTTT  5'-GAACACCTGAATGAAGCAGAAGGCTT |
| ALVE21 | 5'-AAAACCAAACACTTTTGTATATGGGTAGTT  5'-CTGACTTCACTACTCAGCATCACCAA  5'-ACCAAACACTTTTGTATATGGGTAGTG  5'-GAACACCTGAATGAAGCTGAAGGCTT |
| K-duplication | 5'-CCACGGTCCGTGGTTG  5'-ATTGACAGATTGAGAGCTCTTTCTCGATT  5'-ACTAGGGCTAGCATTTAATATAACCCCT  5'-TGAAACCATCCCTGGAGAGATGGAA |

# Table S5: Primers used for the diagnostic Hy-Line-based PCR assays

For each element, the first sequence is the forward, upstream primer and the second is the reverse, downstream primer. In three-primer assays the third primer is the alternative primer, matching the ALVE sequence. Any original Benkel LTR primers are named (LTRA *etc.*). The papers for previously published primers are numbered, where: 1 = Benkel, 1998; 2 = Chang et al., 2006; 3 = Smith & Benkel, 2009; 4 = Smith & Benkel, 2008. References for these four papers are given in the main manuscript.

During testing of all assays, PCR was conducted in 10μl reaction volumes, with equal concentrations of primers, using the Roche FastStart Taq kit. Each PCR reaction began with a 4 minute activation at 95°C, then had 35 cycles of 30 seconds denaturing at 95°C, 30 seconds annealing at 60°C and 45 seconds elongation at 72°C, and then finished with a 7 minute final extension at 72°C. The ALVE15 and ALVEB5 PCR reactions had an annealing temperature of 50°C due to the low primer Tm. In addition, the 45 second elongation within each cycle was extended to 3 minutes for ALVE_ros007.

| Name | Primers | ALVE+ (bp) | ALVE– (bp) |
| --- | --- | --- | --- |
| ALVEB5 | 5'-CAGTCATATATCCGAATGTTTAAGTCT (1)  5'-GGAGCCATAATTTCATAATGAA (1)  5'-CGCCCATATGTCCTTGCGTC (LTRD; 1) | 123 | 241 |
| ALVE1 | 5'-CGGTTATAATGAGGGTTGTGCTTTTC  5'-GCACCAAACAATCTAGTCTGTGC (1)  5'-CCTGAATGAAGCAGAAGGCTTC (LTRA; 1) | 260 | 368 |
| ALVE-ros001 | 5'-TTCCTCTCAGGTCTTCTTGGCG  5'-TGCCCTACGTATGACAATGCTG  5'-AACGATTGCGAACACCTGAATG | 275 | 460 |
| ALVE-ros002 | 5'-TCAGCAGCAACAGAAATCCAGC  5'-CAAGACCCACCTGGATGCCTAC  5'-GGCTATTCAAGTTGCCTCTGGC | 270 | 361 |
| ALVE-ros003 | 5'-TCTCACAAACCCCACAGGTGTC  5'-TTTGTCATCTCTGTGCCCTTGG  5'-GTTGCCTCTGGCTCTATTTGAC | 186 | 498 |
| ALVE-TYR | 5'-TTGAGATACTGGAGGTCTTTAGAAATG (2)  5'-CAAAACCATAAATAGCACTGGAAATAG (2)  5'-CCTCTGGCTCTATTTGACTACACAGT | 345 | 481 |
| ALVE-NSAC1 | 5'-GGTTTGGAGAGCGTTAGCAG (4)  5'-TGACGTCTGTTTTCCCATGA (4)  5'-TGTAGTCAAATAGAGCCAGAGG (LTRC; 1) | 340 | 519 |
| ALVE-ros004 | 5'-CTAAGTAGCTGTCATCCCCACC  5'-AAAGTGTTTCCAGCAGTTTTCC  5'-AAGTTGCCTCTGGCTCTATTTGAC | 273 | 336 |
| ALVE-ros005 | 5'-TGGGGAAGTTGTGCTTTTCCAC  5'-TCTTTGCAACACAGCTTGGGAG | 668 | 388 |
| ALVE-NSAC3 | 5'-TGCTATCTCCCTGCTCATTTG (3)  5'-CACCCAGATCCTTTTCCTCA (3)  5'-GAGTCCCTAACGATTGCGAACAC (LTRF; 1) | 163 | 500 |
| ALVE-ros006 | 5'-TAACTTCTCTTCCAGCCTCAGC  5'-TTTTTCAAGGAGCAGAAAATCC  5'-GAACCCCTAAATGAAGCTGAAG | 333 | 417 |
| ALVE15 | 5'-CAAATGAGGGTAATAAGGGAG (1)  5'-CACTACCAAATATAATTCTGTAG (1) | 460 | 180 |
| ALVE-ros007 | 5'-TCAGCATAAAACCACAGCAAAG  5'-GTGGATTTTGGGCTACTTTCAG | 1,970 | 2,511 |
| ALVE-ros008 | 5'-GCACAGAGAAGGATATGTGCTG  5'-CTGTAAAAGAATCCCATGCCTC  5'-GACTATTCAAGTTGCCTCTGGCTC | 336 | 428 |
| ALVE-ros009 | 5'-ACAGCCTCTCTGGACAACCTGG  5'-GCCCATGTCAAACATCATCAGG  5'-TGTTTTTCCCTTATTTGGTCTTCAG | 248 | 366 |
| ALVE9 | 5'-CATTCTCCATGCACCTGAAGTG (1)  5'-TAGTGCACATATAATTTCAGATGAGTT (1)  5'-ACCTGAATGAAGCTGAAGGCTTC (LTRB; 1) | 115 | 450 |
| ALVE-NSAC7 | 5'-ACACCATCTCCATACACTTCCC  5'-GAAATGCACGTAAGCACAAAAG  5'-TGAATGAAGCAGAAGGCTTCAGAG | 172 | 288 |
| ALVE-ros010 | 5'-CCAAGCTCTGAACATACACTGC  5'-CTGGGTAACAGAAGAGTGGTCC  5'-CTAACGATTGCGAACACCTGAATG | 228 | 433 |
| ALVE3 | 5'-GAAATGCCTGCCCCATGCCAGTG (1)  5'-CTTCTCCAGCTTCAGTGACGC (1)  5'-CCTGAATGAAGCAGAAGGCTTC (LTRA; 1) | 190 | 270 |
| ALVE21 | 5'-CATTTCAAGCAAGGGACTGGC (1)  5'-GTGGGAATGGTACTACAGAGAAGG (1)  5'-ACCTGAATGAAGCTGAAGGCTTC (LTRB; 1) | 390 | 510 |

# Table S6: Generic ALVE sequencing primers.

Positional information was based on the alignment against the ALVE1 reference sequence (GenBank: AY013303.1). Positions shown in Figure S2.

| Name | Sequence | Primer location |
| --- | --- | --- |
| ALV_5LTRrc | 5'-ACCACTATTCCCTAACGATCAC | 290 – 311 |
| ALV_500 | 5'-CGACGACTGAGCAGTCCACCCC | 500 – 521 |
| ALV_1000 | 5'-CGTTGGCACATCCTGCTATCAG | 1,000 – 1,021 |
| ALV_1500 | 5'-TACAGACGGTTATAGCGGCAGC | 1,500 – 1,521 |
| ALV_2000 | 5'-ATCCAGCCCTTAGTTATGGCAG | 2,000 – 2,021 |
| ALV_2500 | 5'-CATGCGAAAATCCCGGGATATG | 2,500 – 2,521 |
| ALV_3000 | 5'-CAAGGATTGCTTCTTTTCTATTC | 3,000 – 3,021 |
| ALV_3500 | 5'-CCTTTTATGAGCAGTTACGAGG | 3,500 – 3,521 |
| ALV_4000rc | 5'-CAGGGTGGTCGGTAACCCTCAC | 3,979 – 4,000 |
| ALV_4500rc | 5'-GGTCTGAACAACCTCCCTAGCC | 4,479 – 4,500 |
| ALV_5000rc | 5'-TCACCACGCTCAAAGTGATTGAG | 4,978 – 5,000 |
| ALV_5500rc | 5'-GAGAGGCAGAAATCCGTTTGGC | 5,479 – 5,500 |
| ALV_6000rc | 5'-ATGCACCGCAGTACTCACTCCC | 5,978 – 6,000 |
| ALV_6500rc | 5'-ATCTGAGCATGTATCATCCAGG | 6,479 – 6,500 |
| ALV_7000rc | 5'-CATCTTTCGGATGCTACTGGAC | 6,983 – 7,004 |
| ALV_3LTR | 5'-GATATAGTAGTTGCGCTTTTGC | 7,215 – 7,236 |

# Table S7: BioNano optic mapping summary statistics.

Optic map statistics for the four analysed Hy-Line samples. Breeds are White Leghorn (WL) and White Plymouth Rock (WPR). Feathering rate is indicated as FF (fast feathering; wildtype) or SF (slow feathering). The consensus map is the total after *de novo* assembly with the average map coverage in brackets. No consensus could be formed for the FF WPR due to limited data, and the other sample consensus maps did not cover the entire Galgal5 assembly (1.2 Gbp). The absence of long contiguous consensus maps limited structural variant detection, and fully contiguous maps were absent across the *K* locus in both SF lines.

| Breed | Feathering | Total molecules | Map N50 (kbp) | Consensus map (Mbp) |
| --- | --- | --- | --- | --- |
| WL | SF | 1,637,620 | 180.1 | 551.0 (39.7X) |
| WL | FF | 1,240,879 | 180.7 | 355.9 (23.7X) |
| WPR | SF | 568,595 | 230.5 | 159.9 (14.8X) |
| WPR | FF | 253,579 | 159.0 | No consensus |
